# Skype interviewing: The new generation of online synchronous interview in qualitative research

**DOI:** 10.3402/qhw.v9.24152

**Published:** 2014-04-15

**Authors:** Roksana Janghorban, Robab Latifnejad Roudsari, Ali Taghipour

**Affiliations:** 1 Student Research Committee, Department of Midwifery, School of Nursing and Midwifery, Mashhad University of Medical Sciences, Mashhad, Iran; 2 Evidence-Based Care Research Center, Department of Midwifery, School of Nursing and Midwifery, Mashhad University of Medical Sciences, Mashhad, Iran. E-mail: LatifnejadR@mums.ac.ir; 3 Health Sciences Research Center, Department of Biostatistics and Epidemiology, School of Health Mashhad University of Medical Sciences, Mashhad, Iran

**Keywords:** Interview, qualitative research, researcher, Skype

## Abstract

The most commonly used method for data collection in qualitative research is interviewing. With technology changes over the last few decades, the online interview has overcome time and financial constraints, geographical dispersion, and physical mobility boundaries, which have adversely affected onsite interviews. Skype as a synchronous online service offers researchers the possibility of conducting individual interviews as well as small focus groups, comparable to onsite types. This commentary presents the characteristics of the Skype interview as an alternative or supplemental choice to investigators who want to change their conventional approach of interviewing.

Interviewing is the most widely used form of data collection in qualitative research (Creswell, 2007). Time and financial constraints, geographical dispersion, and physical mobility boundaries of research populations have presented some problems for conventional face-to-face interviews (Cater, 2011). Over the last few decades, the technological changes in growth of the Internet have developed the experience of online interviewing in qualitative inquiry and have reduced the problems related to face-to-face interviews (Hooley, Wellens, & Marriott, 2012).

Two types of online interviews such as focus group interviews and one-on-one interviews could be performed by both synchronous (real-time) and asynchronous (non-real-time) arenas. Emails, bulletin boards, and discussion groups are the most commonly used methods for asynchronous online interviewing (Hooley et al., 2012). Synchronous approaches focus on text-based chat rooms, instant messenger protocols, and videoconferencing (Stewart & Williams, 2005; Stieger & Gortiz, 2006). Investigators’ interest in using an interactive synchronous method has encouraged them to apply online communication services (Fielding, 2010; Sullivan, 2012). One of the programs released in the past decade is Skype (Anonymous, 2013a).

In 2012, the total number of Skype users was 31 million. However, 560 million people have ever used it (Anonymous, 2012). Skype, as a free communication service, provides the opportunity of calling, seeing, messaging, and sharing with people wherever they are (Anonymous, 2013b). In addition to family, friends, and peer communication, Skype has played various roles in education and research. Educational implications of Skype consist of teaching, learning, and team working on online classrooms (Ryobe, 2008). In its research role, it offers researchers a novel interview method to collect qualitative data (Deakin & Wakefield, 2013).

It provides the opportunity of audio or video interviewing (Anonymous, 2013b). Concurrently interactive communication with direct probing is created in both of them. In using the web camera, the interaction will be comparable to the onsite equivalent for the presence of nonverbal and social cues (Stewart & Williams, 2005; Sullivan, 2012). However, a “head shot” provided by webcam will create obstacles in observing all of the participant's body language (Cater, 2011).

Skype encourages interviewees who have time and place limitations for face-to-face interviews to participate in research. Consequently, the interviews occur in more convenient conditions for participants. The flexibility may resolve the researcher's concern to reach key informants and increase participation. Nevertheless, the selection of a disruptive environment could affect interviewee concentration and data gathering (Deakin & Wakefield, 2013).

Participant recruitment via Skype can be similar to the online and face-to-face interviews. Access to potential participants can be achieved through face-to-face, email, and social networking sites. After participant's agreement to taking part in the study conducted with Skype, the time of the interview will be arranged. This process offers the chance of independent recruitment from traditional gatekeepers, especially in clinical sites. Despite the reported benefits, necessity of access to high-speed Internet, familiarity with online communication, and having digital literacy, affect the nature of the interview (Deakin & Wakefield, 2013; Hamilton & Bowers, 2006).

In Skype interview cases, ethical issues are considered the same as in face-to-face and online interviews. Researchers obtain informed consent by online, email, or posted forms and all participants are fully aware of audio or video recordings. Interviews can be recorded by a separate recorder or computer-based recording software and then transcribed (Cater, 2011; Fox, Morris, & Rumsey, 2007). The online interview gives participants the right to withdraw from the interview process in uncomfortable situations, just by clicking a button. The nature of such communication can increase the absentee rate and rescheduling of interviews compared with face-to-face relationships. However, if this phenomenon occurs, time and financial resources have not been spent (Deakin & Wakefield, 2013).

Access to verbal and nonverbal cues in Skype interviews can provide an equal authenticity level with face-to-face interviews, because the opportunity allows that a visible part of the impression management process can be evaluated (Sullivan, 2012). However, some researchers suggested that “the relative anonymity of online interactions and the lack of a shared social network online” may increase presentation of self and authenticity compared with face-to-face interviews (Bargh, McKenna, & Fitzsimons, 2002; Ellison, Heino, & Gibbs, 2006).

## Conclusion

Skype offers an alternative or supplemental choice to researchers who want to change from conventional face-to-face interviews. It could be used for qualitative research to conduct individual interviews as well as small focus groups. As in each interviewing method, it has some benefits and drawbacks. Thus, before shifting to the new approach in interviewing, researchers need to consider both its advantages and limitations, evaluate its matching level to their research, and then decide whether or not to utilize the method.

*Roksana Janghorban, PhD Student* Student Research Committee Department of Midwifery School of Nursing and Midwifery Mashhad University of Medical Sciences Mashhad, Iran *Robab Latifnejad Roudsari, PhD* Evidence-Based Care Research Center Department of Midwifery School of Nursing and Midwifery Mashhad University of Medical Sciences Mashhad, Iran E-mail: LatifnejadR@mums.ac.ir *Ali Taghipour, PhD* Health Sciences Research Center Department of Biostatistics and Epidemiology School of Health Mashhad University of Medical Sciences Mashhad, Iran
